# Psychological Burnout among Professionals Working with Children with Motor Disabilities

**DOI:** 10.11621/pir.2021.0106

**Published:** 2021-03-31

**Authors:** Taghreed Al-Ali, Mutasem M. Akour, Enas Al-Masri, Annie Abu Hanna Mizaghobian, Soua’d Ghaith

**Affiliations:** a The Hashemite University, Zarqa, Jordan; b Al-Hussein Society for the Rehabilitation of the Physically Challenged, Amman, Jordan

**Keywords:** psychological burnout, emotional exhaustion, depersonalization, children with special needs, Jordan

## Abstract

**Background:**

Psychological burnout is a state of psychological and physical fatigue that shows the effect of work stress on the individual and negatively affects his/her attitudes towards work. The current study was motivated by the assumption that people who work directly with students with special needs are at the forefront of professions that can create feelings of frustration, and thus may be vulnerable to psychological burnout.

**Objective:**

To identify the level of psychological burnout among professionals working with children with motor disabilities, and how these levels differ according to gender, workplace, years of experience, and the number of children the employees treat.

**Design:**

The sample comprised 195 staff members at the Al-Hussein Society for the rehabilitation of the Physically Challenged and the Cerebral Palsy Foundation in Amman, Jordan. The researchers used the Maslach Burnout Inventory, which consists of three dimensions: emotional exhaustion, depersonalization, and the lack of a sense of personal accomplishment.

**Results:**

We found a low level of psychological burnout among those who work with children with motor disabilities. The level of psychological burnout was low for two dimensions: lack of a sense of personal accomplishment and depersonalization, whereas it was average for emotional exhaustion. The results showed statistically significant differences in the degrees of psychological burnout in its three dimensions according to the employee’s workplace, with higher levels at the Cerebral Palsy Foundation. However, no statistically significant differences were found among the participants due to gender, years of experience, or the number of children they treat.

**Conclusions:**

The low levels of psychological burnout among professionals who work with children with motor disabilities might be due to the psychological and professional support they receive from their institutions.

## Introduction

The concept of psychological burnout is of relatively recent origin, having emerged in its modern form during the 1970s in the field of functional psychology. Freudenberger (1974) was the first to introduce this concept to refer to the physical and emotional responses resulting from long-term exposure to work stress among staff at a clinic, who had high and unrealistic expectations. Modern life is full of multiple and varied sources of psychological and mental stress, including from people’s social, psychological, family, professional, marital, political, and academic lives (Ghoneim & Qatanani, 2011). People at work who do not have enough resources to cope with job demands might be in risk for psychological burnout (Seriwatana & Charoensukmongkol, 2020).

Psychological burnout is one of the severe incident to be encountered at work. It is defined as a group of symptoms of nervous stress, exhaustion of emotional energy, depersonalization, and a sense of dissatisfaction with one’s personal achievement in one’s professional field. These symptoms could occur in people who perform work that requires direct interaction with others (Goddard & Goddard, 2006). Psychological burnout is a state of psychological and physical fatigue that shows the effect of work stress on the individual and negatively affects his/her attitudes towards work; it can be clearly diagnosed through the individual’s behavior at work (Askar, 2003).

Maslach(2003) identified three components of psychological burnout: emotional exhaustion, depersonalization, and the loss of a sense of accomplishment. *Emotional exhaustionis* defined as the feeling of being psychologically and mentally exhausted. An emotionally exhausted person feels that he/she no longer wants to work. *Depersonalization* refers to the display of negative attitudes towards everything that relates to work, including coworkers. Lastly, *loss of the sense of accomplishment* indicates lower levels and loss of motivation and self-confidence at work (Angerer, 2003; Kacem et al., 2020). These components were reflected by the instrument used in this study.

Individuals whose jobs require considerable contact with others, such as teachers and health-care personnel, may have a high likelihood of experiencing emotional exhaustion (Chaukos et al., 2017). Previous research has shown that emotional exhaustion can have negative effects both on the employee and the organization (Bakker, Westman, & Schaufeli, 2007). Emotional exhaustion can result in reduced job satisfaction (Shanafelt et al., 2015), inappropriate behavior with coworkers and customers (Kim, 2008), and may lead employees to quit their jobs (Skaalvik & Skaalvik, 2016). In general, psychological burnout may negatively affect employees’ health (Chen & Chen, 2012), job satisfaction, job performance, and organizational commitment (Singh, Goolsby, & Rhoads, 1994).

Efforts have been made in many societies to train specialists to work in the fields of psychological and social services; however, many of these occupations involve various forms of work stress that prevent such specialists from performing their roles effectively, which can weaken their efficiency and ability to help others (Al-Zayoudi, 2007). People working in humanitarian jobs, such as nursing, medicine, counseling, social work, and teaching, are particularly prone to psychological burnout (Keel, 1993). However, those working with people with special needs come at the forefront of professions that can create feelings of frustration. This work requires these professionals to have the skills to deal with various groups of people who suffer from physical, mental, auditory, visual, or multiple disabilities. Each person with a disability is a special case who requires a special pattern of service, learning, training, and assistance. In addition, the diversity and severity of their problems may sometimes create among staff a feeling of frustration and a weak sense of achievement, leading to psychological burnout because of these work-related stressors (Al-Farah, 2001).

The concept of psychological burnout has drawn the attention of many researchers and scholars. In a study conducted by Al-Shami and Al-Smadi (2020) to investigate burnout among teachers at special education institutions in Jordan compared to teachers working in public schools, they found that the level was moderate among all participants regardless of their workplace. There were no significant differences in the level of burnout among teachers according to gender, age, or academic qualification. Similarly, Kharfallah, Balalia, and Sadlib (2019) detected the levels of psychological burnout among 48 people who worked with special needs students. They found that burnout was high, and that those who worked with students who were intellectually disabled suffered from higher levels of burnout than staff who worked with visually impaired and autistic students.

Abu Mustafa and Al-Zein (2009) conducted a study aimed at identifying the sources of work stress among teachers of children with disabilities in special education institutions in Gaza. They found that the most common sources of psychological pressure among teachers were their relationship with the children, their salary, promotion, relationship with colleagues, relationship with the parents, working conditions, relationship with managers, and the work environment.

Platsidou and Agaliotis (2008) studied psychological burnout among teachers of special education in Greece, finding low levels of burnout among teachers on the three dimensions of the Maslach Burnout Inventory (emotional exhaustion, depersonalization, and sense of personal accomplishment). However, no statistically significant differences were found according to the variables of gender and teaching experience.

In another study, Al-Zahrani (2008) tried to identify the nature of the relationship between psychological burnout and some personality traits of staff working with special needs children in the city of Jeddah, Saudi Arabia. The results showed that employees with 11–15 years of experience had more burnout than those with less years of experience. No statistically significant differences in the level of psychological burnout were found according to age, gender, academic qualification, or marital status.

Al-Zayoudi (2007) identified the sources of psychological stress and burnout among teachers of special education in Karak Governorate, Jordan. The subjects suffered from different levels of psychological stress and burnout ranging from medium to high. Male teachers suffered from emotional stress more than female teachers, and teachers with low experience and low income had higher levels of psychological burnout.

In comparing psychological burnout among teachers of ordinary students, gifted students, students with severe learning difficulties, and students with disabilities, Al-Qaryouti and Al-Khatib (2005) found higher levels of burnout for teachers of gifted students and students with severe disabilities as compared to teachers of ordinary students and those with learning difficulties. No statistically significant differences were found according to gender, academic qualification, years of experience, or marital status.

Al-Gamali and Hassan (2003) studied psychological burnout among teachers of students with special needs in the Sultanate of Oman. The results showed that the teachers suffered from a moderate degree of burnout, while teachers who worked with students with multiple disabilities were particularly vulnerable to burnout. No statistically significant differences were found in the three dimensions of psychological burnout according to gender and teaching experience.

Finally, Al-Farah (2001) conducted a study of psychological burnout among teachers working with students with special needs in Qatar, and found a medium degree of burnout. The results also showed that specialists in the treatment and training of people with special needs were more vulnerable to burnout than the two categories of teachers in the field of special education. There were no statistically significant differences in the level of psychological burnout due to educational level or years of experience. Teachers who work with students with multiple disabilities suffered from depersonalization more than those who work with students with mental disabilities and physical sensory disabilities.

### Research Questions

Psychological burnout and its consequences leave negative impacts on the individual, whether in relation to adaptation to or controlling any challenges, which may also extend to include others who interact and communicate with this individual. People who work directly with students with special needs, such as teachers and specialists, or others such as administrators, are at the forefront of professions that can create feelings of frustration. Therefore, the current study aimed at revealing the level of psychological burnout among staff who work with people with special needs and motor disabilities in the relevant centers in Amman, Jordan. We also sought to identify the level of psychological burnout among staff in the field of motor disability according to different variables. More specifically, the current study attempted to answer the following research questions:

1. What is the level of psychological burnout among staff who work with children with motor disabilities?
2. Are there statistically significant differences in the level of psychological burnout among those who work with children with motor disabilities, according to the gender of the staff member?
3. Are there statistically significant differences in the level of psychological burnout among those who work with children with motor disabilities, according to the workplace?
4. Are there statistically significant differences in the level of psychological burnout among those who work with children with motor disabilities, according to their years of experience?
5. Are there statistically significant differences in the level of psychological burnout among those who work with children with motor disabilities, according to the number of children they treat?

### Significance of the Study

Working with persons with special needs is one of the professions most exposed to psychological burnout. This study is one of the few that has tackled this phenomenon among teachers, specialists, and administrators who work with children with motor disabilities in Jordan. Children with different types of disability require a special pattern of service, learning, training, and assistance. Therefore, it is of importance to compare burnout among professionals who deal with children with different types of disabilities. The findings of the current study might suggest the need to develop appropriate counseling programs for those who are dealing with children with different types of disabilities, to support them in reducing psychological burnout, and thus to achieve psychological adaptation that would result in better performance. The psychological status of these workers, if stable and controlled, will improve the adaptation and performance of children with motor disabilities academically, developmentally, and socially.

## Method

### Sampling

The targeted population for this study consisted of all professional employees (N = 215) at Al-Hussein Society for the rehabilitation of the Physically Challenged , and at the Cerebral Palsy Foundation, in Amman, Jordan. These two institutions are specialized centers concerned with motor disability, and work under the Supreme Council for Persons with Disabilities in Jordan. The professional employees in these two institutions include: teachers, administrators, specialists, physiotherapists, occupational therapists, doctors, and nurses. The sample comprised 195 staff members who volunteered to participate. Of these, 129 (66%) were females, and 66 were males. Of the study sample, 109 (56%) people work at the Al-Hussein Society and the remaining work at the Cerebral Palsy Foundation.

### Instrument

The instrument of this study consisted of two parts. The first part contains demographic information about the sample, such as: gender, workplace, years of experience, and the number of children the employee treats. The second part consists of the Maslach Burnout Inventory (Maslach & Jackson, 1981), which was used to identify the levels of psychological burnout. This inventory is widely used as a global scale for psychological burnout and has been used in many Arabic and other international studies (Al-Farah, 2001; Al-Gamali & Hassan, 2003; Al-Kharabsheh, 2005; Bataineh & Al-Jawarneh, 2004; Schwarzer & Hallum, 2008; Worley, Vassar, Wheeler, & Barnes, 2008).

The inventory consists of 22 items related to the individual’s feeling towards the profession. Each participant is asked to respond twice to each item: the first response indicating a repeat of the feeling (scored on a scale from 0 to 6), and the second one indicating the intensity of feeling (scored on a scale from 0 to 7). These items are divided into three dimensions: (a) emotional exhaustion: item numbers 1, 2, 3, 6, 8, 13, 14, 16, and 20; (b) depersonalization: items numbers 5, 10, 11, 15, and 22; and (c) lack of a sense of personal accomplishment: item numbers 4, 7, 9, 12, 17, 18, 19, and 21.

The score of each participant was calculated for each item, on the three dimensions of the Maslach Burnout Inventory (emotional exhaustion [EE], depersonalization [DP], and lack of a sense of personal accomplishment [PA]), and on the total scale. In interpreting the scores, the following scale was used: 1–2.66 (low), 2.67–4.33 (medium), and 4.34–6 (high).

Reliability of the scores was estimated using Cronbach’s alpha, which was 0.85 for the whole scale. Corrected item-total correlations were computed as a measure of the internal consistency of the instrument as provided in Table 1. All values ranged between 0.23 for item 15 and 0.60 for item 8. All corrected item-total correlations exceeded that value of 0.2, which indicated that all items were consistent in measuring psychological burnout.

**Table 1:** Means, standard deviations, and levels of psychological burnout for scores on the burnout scale and its three dimensions

Dimension	Mean	SD	Level of burnout
Emotional exhaustion	2.81	0.80	Medium
Depersonalization	2.32	0.85	Low
Lack of a sense of personal accomplishment	2.36	0.85	Low
Total scale	2.54	0.71	Low

### Data Analysis

To answer our first research question, we computed the mean and standard deviation for each item, for each dimension, and for the total scale. The t-test and one-way ANOVA were used to detect differences in psychological burnout among those working with children with motor disabilities, according to gender, workplace, experience, and number of children they treat.

## Results

The findings are presented for each research question sequentially.

### First Research Question

To find the level of psychological burnout among staff working with children with motor disabilities, the mean and standard deviation for each dimension and for the total scale were computed and are presented in *Table 1*.

*Table 1* shows that in general, the level of psychological burnout among those working with children with motor disability was low (mean = 2.54, SD = 0.71). The levels of depersonalization and lack of a sense of personal accomplishment were also low (mean = 2.32, SD = 0.85, and mean = 2.36, SD = 0.85, respectively). The level of emotional exhaustion was medium (mean = 2.81, SD = 0.80).

To have a better visualization of these results, the mean and standard deviation for participants’ score on each item were computed and are presented in *Tables 2*, *3*, and *4*.

**Table 2:** Means and standards deviations for scores on each item related to emotional exhaustion

Item	Mean	SD	Level
2- I feel used up at the end of the workday.	3.67	1.80	Medium
16- I deal very effectively with employees’ problems.	3.44	1.80	Medium
22- I deal with the emotional problems of employees during my professional practice.	3.23	1.85	Medium
6- I feel overwhelmed by the practice of this profession.	2.93	1.73	Medium
1- I feel emotionally drained from practicing this profession.	2.66	1.70	Medium
13- Actually, I don’t care or take interest in the problems of employees.	2.58	1.78	Low
3- I feel anxious when I wake up and know that I will have to face new work.	2.45	1.77	Low
8- Working directly with people causes severe emotional stress.	2.24	1.54	Low
14- I feel that employees blame me for some of the problems they face.	2.14	1.54	Low

**Table 3:** Means and standards deviations for scores on each item related to depersonalization

Item	Mean	SD	Level
15- I can easily understand employees’ feelings about things.	2.97	1.71	Medium
20- I feel happy and comfortable after working with the employees.	2.73	1.75	Medium
5- I feel psychological exhaustion from practicing this profession.	2.43	1.66	Low
10- I feel like I am dealing with some of the employees as if they were not human.	1.75	1.43	Low
11- I became cruel with people after joining this profession.	1.71	1.33	Low

**Table 4:** Means and standards deviations for scores on each item related to lack of a sense of personal accomplishment

Item	Mean	SD	Level
7- I feel that I work in this profession under great stress.	3.18	1.866	Medium
18- I feel active and energetic.	2.64	1.716	Low
4- Dealing with people all day long causes stress and fatigue.	2.58	1.686	Low
19- I can easily create a comfortable psychological atmosphere with the employees.	2.44	1.674	Low
17- I feel that I have a positive impact on the lives of many people through practicing this profession.	2.36	1.667	Low
21- I have accomplished many valued and important things in this profession.	2.25	1.650	Low
12- I feel disturbed and anxious because this profession increases the cruelty of my emotions.	1.75	1.352	Low
9- I feel like I’m at the edge of desperation from practicing this profession.	1.64	1.262	Low

*Table 2* shows that study participants had low levels of emotional exhaustion on half of the items (items 13, 3, 8, and 14), with item means ranging between 2.14 for item 14 (I feel that employees blame me for some of the problems they face) to 2.58 for item 13 (Actually, I don’t care or take interest in the problems of employees). Medium levels of emotional exhaustion were captured on the other half of the items (items 2, 16, 22, 6, and 1), with item means ranging between 2.66 for item 1 (I feel emotionally drained from practicing this profession ) to 3.67 for item 2 (I feel used up at the end of the workday).

*Table 3* shows that people working with children with disabilities had low levels of depersonalization on three items (items 5, 10, and 11), with item means ranging between 1.71 for item 11 (I became cruel with people after joining this profession) to 2.43 for item 5 (I feel psychological exhaustion from practicing this profession). Levels of depersonalization on the remaining two items (items 15 and 20) were medium, with the item mean of 2.73 for item 20 (I feel happy and comfortable after working with the employees) and 2.97 for item 15 (I can easily understand employees’ feelings about things).

*Table 4* shows that the level of lack of a sense of personal accomplishment among those working with children with motor disability was low for all items, except for item 7 (I feel that I work in this profession under great stress), which was medium. The level of psychological burnout was low for items 18, 4, 19, 17, 21, 12, and 9.

### Second Research Question

To find out whether the level of psychological burnout differs between male and female staff working with children with motor disabilities, an independent samples t-test was carried out on each dimension score and on the total scale scores, as presented in Table 5. The assumption of equality of variances was checked via Levene’s test. It was found that variances of the scores for males and females were homogeneous across each dimension.

**Table 5:** The t-test for the psychological burnout scale and its three dimensions as a function of staff members’ gender

Dimension	Gender	N	Mean	SD	t
Emotional exhaustion	Male	66	2.76	0.83	0.66
	Female	129	2.84	0.79	
Depersonalization	Male	66	2.38	0.85	0.78
	Female	129	2.28	0.86	
Lack of a sense of personal accomplishment	Male	66	2.41	0.83	0.70
	Female	129	2.32	0.86	
Total scale	Male	66	2.55	0.73	0.21
	Female	129	2.53	0.71	

*Table 5* shows that there were no statistically significant differences in the level of psychological burnout between male and female staff working with children with motor disabilities, t = 0.21, p > 0.05. In addition, no statistically significant differences in the level of psychological burnout between male and female staff working with children with motor disabilities were found on the dimensions of emotional exhaustion (t = 0.66, p > 0.05), depersonalization (t = 0.78, p > 0.05), and lack of a sense of personal accomplishment (t = 0.70, p > 0.05).

This indicates that the levels of psychological burnout and the levels of emotional exhaustion, depersonalization, and lack of a sense of personal accomplishment were the same for both male and female staff working with children with motor disabilities.

### Third Research Question

To find out whether the level of psychological burnout differs among staff working with children with motor disabilities according to their workplace, an independent samples t-test was carried out on each dimension score and on the total scale scores, as presented in Table 6. The assumption of equality of variances was checked via Levene’s test. It was found that variances of the scores for workers at the two workplaces were homogeneous across each dimension.

**Table 6:** The t-test for the psychological burnout scale and its three dimensions as a function of workplace

Dimensions	Workplace	N	Mean	SD	t
Emotional exhaustion	Al-Hussein Society	109	2.66	0.74	3.04**
	Cerebral Palsy Foundation	86	3.01	0.83	
Depersonalization	Al-Hussein Society	109	2.19	0.78	2.30*
	Cerebral Palsy Foundation	86	2.47	0.92	
Lack of a sense of personal accomplishment	Al-Hussein Society	109	2.23	0.77	2.37*
	Cerebral Palsy Foundation	86	2.52	0.92	
Total scale	Al-Hussein Society	109	2.40	0.65	3.06**
	Cerebral Palsy Foundation	86	2.71	0.76	

Note. * p < 0.05. ** p < 0.01

Table 6 shows that there were statistically significant differences in the level of psychological burnout among staff working with children with motor disabilities according to their workplace, t = 3.06, p < 0.01. Staff at the Cerebral Palsy Foundation tended to have higher levels of psychological burnout as compared to those at the Al-Hussein Society. Moreover, workers at the Cerebral Palsy Foundation, as compared to workers at Al-Hussein Society, had statistically significant higher levels of emotional exhaustion (t = 3.04, p < 0.01), depersonalization (t = 2.30, p < 0.05), and lack of a sense of personal accomplishment (t = 2.37, p < 0.5).

### Fourth Research Question

To find out whether the level of psychological burnout differs among staff working with children with motor disabilities according to their years of experience, the mean and standard deviation were computed for each dimension score and for the total scale scores, as presented in *Table 7*.

**Table 7:** Means and standards deviations for score on the burnout scale and its three dimensions as a function of years of experience

Dimension	Years of experience	N	Mean	SD
Emotional exhaustion	Less than 5 years	64	2.94	0.85
	From 5 to 9 years	43	2.75	0.64
	From 10 to 14 years	36	2.93	1.01
	15 years and over	52	2.63	0.68
Depersonalization	Less than 5 years	64	2.33	0.87
	From 5 to 9 years	43	2.35	0.87
	From 10 to 14 years	36	2.51	0.91
	15 years and over	52	2.14	0.76
Lack of a sense of personal accomplishment	Less than 5 years	64	2.39	0.74
	From 5 to 9 years	43	2.47	0.99
	From 10 to 14 years	36	2.42	0.99
	15 years and over	52	2.16	0.72
Total scale	Less than 5 years	64	2.61	0.69
	From 5 to 9 years	43	2.56	0.73
	From 10 to 14 years	36	2.65	0.88
	15 years and over	52	2.35	0.59

*Table 7* shows that the mean of the scores on the burnout scale and its three dimensions as a function of years of experience ranged from 2.14 to 2.94. In order to examine the statistical significance of these differences, a one-way ANOVA was conducted, and the results are presented in Table 8.

**Table 8:** One-way ANOVA for the psychological burnout scale and its three dimensions as a function of staff members’ years of experience

Dimension	Source of variance	SS	df	MS	F
Emotional exhaustion	Years of experience	3.431	3	1.14	1.80
	Error	121.347	191	.64	
	Total	124.778	194		
Depersonalization	Years of experience	3.014	3	1.01	1.39
	Error	138.073	191	.72	
	Total	141.087	194		
Lack of a sense of personal accomplishment	Years of experience	2.873	3	.96	1.33
	Error	137.441	191	.72	
	Total	140.314	194		
Total scale	Years of experience	2.647	3	.88	1.74
	Error	96.649	191	.51	
	Total	99.297	194		

*Table 8* shows that there were no statistically significant differences in the level of psychological burnout as a function of years of experience (F = 1.74, p > 0.05). In addition, no statistically significant differences were found as a function of years of experience with respect to emotional exhaustion (F = 1.80, p > 0.05), depersonalization (F = 1.39, p > 0.05), and lack of a sense of personal accomplishment (F = 1.33, p > 0.05). This indicates that the level of psychological burnout, emotional exhaustion, depersonalization, and lack of a sense of personal accomplishment did not differ according to the staff members’ years of experience.

### Fifth Research Question

To find out whether the level of psychological burnout differs among those working with children with motor disabilities according to the number of children they treat, means and standard deviations were computed for each dimension score and for the total scale scores, as presented in *Table 9*.

**Table 9:** Means and standards deviations for scores on the burnout scale and its three dimensions as a function of the number of children the staff member treats

Dimensions of burnout	No. of children	N	Mean	SD
Emotional exhaustion	< 15	42	2.97	0.78
	15 – 19	34	2.65	0.64
	20 – 25	22	2.90	0.92
	> 25	97	2.79	0.83
Depersonalization	< 15	42	2.37	1.05
	15 – 19	34	2.28	0.75
	20 – 25	22	2.08	0.85
	> 25	97	2.37	0.80
Lack of a sense of personal accomplishment	< 15	42	2.54	0.94
	15 – 19	34	2.33	0.77
	20 – 25	22	2.31	0.82
	> 25	97	2.29	0.85
Total scale	< 15	42	2.68	0.80
	15 – 19	34	2.45	0.60
	20 – 25	22	2.50	0.75
	> 25	97	2.51	0.71

*Table 9* shows that the mean of the scores on the burnout scale and its three dimensions as a function of years of experience ranged from 2.08 to 2.97. To examine the statistical significance of these differences, a one-way ANOVA was conducted, and the results are presented in *Table 10*.

**Table 10:** One-way ANOVA for the psychological burnout scale and its three dimensions as a function of the number of children the employee treats

Dimension	Source	SS	df	MS	F
Emotional exhaustion	No. of children	2.193	3	.731	1.14
	Error	122.585	191	.642	
	Total	124.778	194		
Depersonalization	No. of children	1.599	3	.533	0.73
	Error	139.488	191	.730	
	Total	141.087	194		
Lack of a sense of personal accomplishment	No. of children	1.907	3	.636	0.88
	Error	138.406	191	.725	
	Total	140.314	194		
Total scale	No. of children	1.191	3	.397	0.77
	Error	98.105	191	.514	
	Total	99.297	194		

*Table 10* shows that there were no statistically significant differences in the level of psychological burnout as a function of the number of children the employee treats (F = 0.77, p > 0.05). In addition, no statistically significant differences were found as a function of the number of children the employee treats with respect to emotional exhaustion (F = 1.14, p > 0.05), depersonalization (F = 0.73, p > 0.05), and lack of a sense of personal accomplishment (F = 0.88, p > 0.05). This indicates that the level of psychological burnout, emotional exhaustion, depersonalization, and lack of a sense of personal accomplishment did not differ according to the number of children the employee treats.

## Discussion

This study tackled the issue of psychological burnout among professional staff working with children in the field of motor disability in Jordan, and its relationship to some demographic variables. The exposure of many working in the human and social professions to psychological burnout motivated the study.

The results showed that staff working with children with motor disabilities did not suffer from psychological burnout. The levels of psychological burnout, depersonalization, and lack of a sense of personal accomplishment were low, although the level of emotional exhaustion was medium. These findings agree with those of Platsidou and Agaliotis (2008), which showed that Greek teachers had low levels of psychological burnout on the three dimensions of the scale.

On the other hand, the results of the current study disagree with those of Al-Farah (2001), which showed that psychological burnout among those working with students with special needs in Qatar was at a medium level. In addition, the results of this study disagree with those of Al-Zayoudi (2007), which showed that special education teachers in the Karak Governorate were subject to psychological burnout ranging from medium to high.

The low levels of psychological burnout among the sample of this study could be attributed to the type of disability that those working in this field in Amman confront – motor disabilities–whereas the previous studies were conducted with teachers who work with people with mental, hearing, visual or multiple disabilities.

The present study also found no statistically significant differences between males and females in the level of psychological burnout with its three dimensions among those working with children with motor disabilities. This result is consistent with previous studies (Al-Gamali & Hassan, 2003; Al-Qaryouti & Al-Khatib, 2005; Platsidou & Agaliotis, 2008). However, this result disagreed with those of Al-Farah (2001), which indicated that males working with people with special needs are more vulnerable to psychological burnout than females. In the current study, the researchers noted that some male staff members themselves have a motor disability. This could explain the absence of statistically significant differences in the level of psychological burnout in the study sample due to the employees’ gender.

According to the workplace, the results of the present study indicated that the level of psychological burnout with its three dimensions was higher at the Cerebral Palsy Foundation than at the in Al-Hussein Society. This result may be attributed to the fact that the Cerebral Palsy Foundation deals with other types of disability, particularly mental disability, in addition to physical disability, while the Al-Hussein Society deals only with people with motor disabilities.

The results also found no statistically significant differences in the degree of psychological burnout with its three dimensions according to the employees’ years of experience. This result is consistent with previous studies (Al-Farah, 2001; Al-Gamali & Hassan, 2003). However, the results disagree with those of Al-Zayoudi (2007), which indicated that employees with less experience were more exposed to psychological burnout than those with more experience. In addition, the results of this study did not go along with those of Al-Zahrani (2008), which indicated that female teachers with 11–15 years of experience were more exposed to psychological burnout than were other categories of staff.

The present study found no statistically significant differences in the degree of psychological burnout with its three dimensions among staff working with children with motor disabilities attributed to the number of children the employee treats. Miller, Brownell, and Smith(1999) indicated that one of the reasons that teachers stop working with people with disabilities is the large number of students. This is not consistent with the results of the present study, which did not show any statistically significant difference in the level of burnout due to the number of children.

## Conclusions

The findings of the present study revealed that the teachers working with children with motor disabilities had low levels of psychological burnout. These levels did not differ as a function of the teachers’ gender, years of experience, or the number of children they treat. This may be a result of the psychological and professional support that these educational and service institutions provide their employees who serve children with motor disabilities. It may also indicate the amount of continuous support through rehabilitation and selection of the best qualified employees to work in supporting these children. It is hoped that centers that work with children with motor disabilities will extend such support and contribute to alleviating the suffering of these children and their parents.

## Limitations

One of the limitations of the current study was related to the sample, which involved only one type of disability, namely motor disability. Future research could study psychological burnout among those working with children with other types of disabilities, in addition to examining the effects of other variables on psychological burnout, such as educational qualifications, personal characteristics, and monthly income.

## References

[ref1] Abu Mustafa N., & Al-Zein, D. (2009). Job Stress Sources among Special Education Teachers. Journal of the Islamic University, 17 (2), 303–347.:10.33976/iugjhr.v17i2.919

[ref2] Al-Farah, A. (2001). Psychological burnout among workers with persons with special needs in the State of Qatar. Dirasat: Educational Sciences, 28 (2), 247–271.

[ref3] Al-Gamali, F., & Hassan, A. (2003). Levels of psychological burnout among teachers with special needs and their training needs in the Sultanate of Oman. Arab Studies in Psychology, 2 (1), 151–211.

[ref4] Al-Kharabsheh, O. (2005). Levels of psychological burnout among educational counselors in public schools in Jordan (Unpublished master’s thesis). Yarmouk University, Irbid, Jordan.

[ref5] Al-Qaryouti, I., & Al-Khatib, F. (2005). Psychological burnout among teachersof normal students and students with special needs. Journal of the Faculty of Education–UAE University, 23 , 131–154.

[ref6] Al-Shami, K. & Al-Smadi, S. (2020.) The level of burnout among special education teachers compared to teachers working in public school in the province of Irbid in relationship to some variables. Journal of Education/Al Mejlh Altrbwyh, 2 (34), 189–226.

[ref7] Al-Zahrani, N. (2008). Psychological burnout and its relationship to some personality traits among workers with special needs (Unpublished master’s thesis). Umm Al-Qura University, Mecca, Saudi Arabia.

[ref8] Al-Zayoudi, M. (2007). Sources of psychological stress and psychological burnout among special education teachers in Karak Governorate and their relationship to some variables. Damascus University Journal of Educational and Psychological Sciences, 23 (2), 189–219.

[ref9] Angerer, J. M., (2003). Job burnout. Journal of Employment Counseling, 40 , 98–107.https://doi10.1002/j.2161-1920.2003.tb00860.x:

[ref10] Askar, A. (2003). Psychological and social foundations of behavior in the field of work . Cairo: Dar Al-Kitab Al-Hadith.

[ref11] Bakker, A. B., Westman, M., & Schaufeli, W. B. (2007). Crossover of burnout: An experimental design. European Journal of Work and Organizational Psychology, 16 (2), 220–239.:10.1080/13594320701218288

[ref12] Bataineh, O., & Al-Jawarneh, A. (2004). The levels of psychological burnout among special education teachers in Irbid Governorate and their relationship to some variables. Journal of the Federation of Arab Universities for Education and Psychology, 2 (2), 46–76.

[ref13] Chaukos, D., Chad-Friedman, E., Mehta, D. H., Byerly, L., Celik, A., McCoy, T.H. & Denninger, J. W. (2017). Risk and resilience factors associated with resident burnout. Academic Psychiatry, 41 (2), 189–194.https://doi:10.1007/s40596-016-0628-6 28028738 10.1007/s40596-016-0628-6

[ref14] Chen, C. F., & Chen, S. C. (2012). Burnout and work engagement among cabin crew: Antecedents and consequences. The International Journal of Aviation Psychology, 22 (1), 41–58.

[ref15] Freudenberger, H. J. (1974). Staff Burn-Out. Journal of Social Issues, 30(1), 159–165. 10.1111/j.1540-4560.1974.tb00706.x

[ref16] Ghoneim, K., & Qatanani, H. (2011). Psychologicalburnout of a sample of psychological counselors in public schools in Balqa Governorate and its association with some variables. Journal of the Faculty of Education-Ain Shams University, 35 (2), 221–265.

[ref17] Goddard, R., & Goddard, M. (2006). Beginning teacherburnout in Queensland schools: Associations with serious intentions to leave. The Australian Educational Researcher, 33 (2), 61–75.https://doi:10.1007/BF03216834

[ref18] Kacem, I., Kahloul, M., Arem, S., Ayachi, S., Hafsia, M., Maoua, M., Ben Othmane, M.,… & Mrizek, N. (2020). Effects of music therapy on occupational stress and burn-out risk of operating room staff.. Libyan Journal of Medicine, 15(1), 1768024.https://doi10.1080/19932820.2020.1768024:. 10.1080/19932820.2020.1768024 PMC7448868 32449482

[ref19] Keel, P. (1993). Psychological stress caused by work: Burnout syndrome. Soz Praventivmed, 38 Suppl 2 , 131–132.https://doi:10.1007/bf01305364 10.1007/BF01305364 8279188

[ref20] Kharfallah, A. Balalia, M., & Sadlib, J. (2019). Psychological burnout among workers with some special needs groups in the light of two variables. Journal of Psychological & Educational Sciences, 5 (2), 156–173.

[ref21] Kim, H.J. (2008). Hotel service providers’ emotional labor: The antecedents and effects on burnout. International Journal of Hospitality Management, 27 (2), 151–161.https://doi:10.1016/j.ijhm.2007.07.019

[ref22] Maslach, C. (2003). Job burnout: New directions in research and intervention. Current Directions in Psychological Science , 12 (5), 189–192.https://doi:10.1111/1467-8721.01258

[ref23] Maslach, C., & Jackson, S. E. (1981). The measurement of experienced burnout. Journal of Organizational Behavior, 2 (2), 99–113.https://doi:10.1002/job.4030020205

[ref24] Miller, M. D., Brownell, M. T., & Smith, S. W. (1999). Factors That Predict Teachers Staying in, Leaving, or Transferring from the Special Education Classroom. Exceptional Children , 65 (2), 201–218. 10.1177/001440299906500206

[ref25] Platsidou, M., & Agaliotis, I. (2008). Burnout, job satisfactionand instructional assignment-related sources of stress in Greek special education teachers. International Journal of Disability Development and Education, 55 (1), 61–76.https://doi:10.1080/10349120701654613

[ref26] Seriwatana, P., & Charoensukmongkol, P. (2020). The effect of cultural intelligence on burnout of Thai cabin crew in non-national airlines moderated by job tenure. ABAC Journal, 40 (1), 1–19.

[ref27] Schwarzer, R., & Hallum, S. (2008). Perceived teacher self-efficacy as a predictor of job stress and burnout: Mediation analyses. Applied Psychology, 57 (s1), 152–171.https://doi:10.1111/j.1464-0597.2008.00359.x

[ref28] Shanafelt, T. D., Hasan, O., Dyrbye, L. N., Sinsky, C., Satele, D., Sloan, J. & West, C. P. (2015). Changes in burnout and satisfaction with work–life balance in physicians and the general US working population between 2011 and 2014. Mayo Clinic Proceedings, 90, (12), 1600–1613.https://doi:10.1016/j.mayocp.2015.08.023 26653297 10.1016/j.mayocp.2015.08.023

[ref29] Singh, J., Goolsby, J. R., & Rhoads, G. K. (1994). Behavioral and psychological consequences of boundary spanning burnout for customer service representatives. Journal of Marketing Research, 31 (4), 558–569.https://doi:10.1177/002224379403100409

[ref30] Skaalvik, E., M. & Skaalvik, S. (2016). Teacher stress and teacher self-efficacy as predictors of engagement, emotional exhaustion, and motivation to leave the teaching profession. Creative Education, 7 (1), 1785–1799.https://doi:10.4236/ce.2016.713182

[ref31] Worley, J. A., Vassar, M., Wheeler, D. L., & Barnes, L. L. B. (2008). Factor structure of scores from the Maslach Burnout Inventory: A review and meta-analysis of 45 exploratory and confirmatory factor-analytic studies. Educational and Psychological Measurement, 68 (5), 797–823.https://doi:10.1177/0013164408315268

